# BH3 mimetics induce apoptosis independent of DRP-1 in melanoma

**DOI:** 10.1038/s41419-018-0932-z

**Published:** 2018-09-05

**Authors:** Nabanita Mukherjee, Andrew Strosnider, Bay Vagher, Karoline A. Lambert, Sarah Slaven, William A. Robinson, Carol M. Amato, Kasey L. Couts, Judson G. T. Bemis, Jacqueline A. Turner, David A. Norris, Yiqun G. Shellman

**Affiliations:** 1 University of Colorado Anschutz Medical Campus, School of Medicine, Department of Dermatology, Mail Stop 8127, Aurora, CO 80045 USA; 2 University of Colorado Anschutz Medical Campus, School of Medicine, Division of Medical Oncology, Mail Stop 8117, Aurora, CO 80045 USA; 3 Department of Veterans Affairs Medical Center, Dermatology Section, Denver, CO 80220 USA; 4 University of Colorado Anschutz Medical Campus, Gates Center for Regenerative Medicine, Aurora, CO 80045 USA

## Abstract

Despite the recent advancement in treating melanoma, options are still limited for patients without *BRAF* mutations or in relapse from current treatments. BH3 mimetics against members of the BCL-2 family have gained excitement with the recent success in hematological malignancies. However, single drug BH3 mimetic therapy in melanoma has limited effectiveness due to escape by the anti-apoptotic protein MCL-1 and/or survival of melanoma-initiating cells (MICs). We tested the efficacy of the BH3 mimetic combination of A-1210477 (an MCL-1 inhibitor) and ABT-263 (a BCL-2/BCL-XL/BCL-W inhibitor) in killing melanoma, especially MICs. We also sought to better define Dynamin-Related Protein 1 (DRP-1)’s role in melanoma; DRP-1 is known to interact with members of the BCL-2 family and is a possible therapeutic target for melanoma treatment. We used multiple assays (cell viability, apoptosis, bright field, immunoblot, and sphere formation), as well as the CRISPR/Cas9 genome-editing techniques. For clinical relevance, we employed patient samples of different mutation status, including some relapsed from current treatments such as anti-PD-1 immunotherapy. We found the BH3 mimetic combination kill both the MICs and non-MICs (bulk of melanoma) in all cell lines and patient samples irrespective of the mutation status or relapsed state (*p* < 0.05). Unexpectedly, the major pro-apoptotic proteins, NOXA and BIM, are not necessary for the combination-induced cell death. Furthermore, the combination impedes the activation of DRP-1, and inhibition of DRP-1 further enhances apoptosis (*p* < 0.05). DRP-1 effects in melanoma differ from those seen in other cancer cells. These results provide new insights into BCL-2 family’s regulation of the apoptotic pathway in melanoma, and suggest that inhibiting the major anti-apoptotic proteins is sufficient to induce cell death even without involvement from major pro-apoptotic proteins. Importantly, our study also indicates that DRP-1 inhibition is a promising adjuvant for BH3 mimetics in melanoma treatment.

## Introduction

The BCL-2 family of proteins plays a crucial role in regulating cell death. Upregulation of anti-apoptotic BCL-2 family members contribute to tumorigenesis, and resistance to chemotherapy and molecular-targeted therapies^1,2^. BH3 mimetics represent a novel class of cancer therapeutic drugs, which directly activate apoptosis by binding and inhibiting anti-apoptotic BCL-2 family members, thus bypassing the requirement for upstream initiators, such as p53^3^. BH3 mimetics have recently generated a lot of excitement for cancer treatments, and multiple drugs are currently in clinical trials, including Navitoclax/ABT-263 (a small-molecule BCL-2/BCL-XL/BCL-W inhibitor)^4–7^. In early clinical trials, an oil-based liquid formulation of ABT-263 showed promising result in lymphoma, but was accompanied with dose-limiting thrombocytopenia^8^. Recent improvement in its formulation and dosage management has reduced the drug-induced cytotoxicity^9^. Currently, it is in clinical trials for small cell lung cancer, acute lymphoblastic leukemia, and other solid tumors, including melanoma^10–13^. Apart from its anti-cancer effect, ABT-263 has also been shown to kill senescent cells^14^, generating excitement in rejuvenation and anti-aging research through targeting BCL-2 family members.

Melanoma is a devastating cancer. The treatments for melanoma have dramatically advanced in the recent years, and currently include targeted therapies against BRAF or MEK, and immunotherapy drugs. However, targeted therapies only work on a subset of patients with particular mutations, and almost all relapse eventually. Although promising, immunotherapies are not without caveats—not all patients respond, some patients experience relapse, and immune-related side effects are a serious concern. For instance, the most promising immune therapy, PD-1 inhibitors, are reported to have 30–40% initial response rate in melanoma^15^, but approximately 25% of melanoma patients had recurrent disease in less than 2 years^16^. Thus, alternative treatment options for melanoma are still needed.

Relapse is partly attributed to the presence of melanoma-initiating cells (MICs)^17–23^, stem-like cells often resistant to chemotherapeutics. Many studies demonstrate that several anti-apoptotic BCL-2 family members need to be targeted to kill various solid tumors; particularly MCL-1 has emerged as a fundamental target^24,25^. MCL-1 is an important anti-apoptotic protein of the BCL-2 family^1,2^, whose upregulation is often associated with chemotherapeutic escape^26^. Pharmacological inhibition or downregulation of MCL-1 promotes apoptosis and/or overcome drug resistance in multiple cancers, including melanoma, making it a high-priority therapeutic target^27–30^. However, we have previously found that along with MCL-1, other BCL-2 family of anti-apoptotic proteins need to be inhibited to kill both non-MICs and MICs^19,21,22^.

The generation of an effective and selective small-molecule MCL-1 inhibitor has been a challenge; however, recently A-1210477 has emerged as an effective and potent inhibitor^31^. It induces rapid apoptosis in MCL-1-dependent cell lines^32^, and significantly sensitizes a variety of solid tumor cell lines to ABT-263 (ref.^31^). In several cancers, cell death induced by A-1210477 is dependent on a mitochondrial protein DRP-1, and inhibition of DRP-1 protected the cells from A-1210477 or combination induced cell death^32^. However, it has not been tested whether and how DRP-1 inhibition may affect treatment of the BH3 mimetics in melanoma specifically.

Here, we examined whether the potent MCL-1 inhibitor A-1210477, in combination with ABT-263, kills the MIC and non-MIC population of melanoma cell lines and relapsed patient samples. The term “de-bulk” is used in this manuscript to describe the targeting of non-MIC population. A diverse array of patient samples was used, with various mutations such as *BRAF, NRAS*, or *BRAF*-Fusion, as well as a sample of triple-WT (wild type for *BRAF*, *NRAS*, and *NF-1*). Some of the samples used in the study have relapsed from BRAF/MEK inhibitors or anti-PD-1 immunotherapy treatments (Supplementary Table 1). We employed multiple assays to dissect the effects of the combination on the MIC and non-MIC populations, and used shRNA and CRISPR/Cas9 techniques for mechanistic studies. Further, we compared the dependence on DRP-1 in melanoma versus breast cancer cells, which are known for their dependence. Our studies suggest that targeting multiple BCL-2 family members, with and without inhibiting DRP-1, is worthy of exploring further in melanoma treatment. This includes melanomas unresponsive or relapsed from the current targeted or immunotherapies.

## Materials and methods

### Reagents and drug treatments

A-1210477 (99.59% purity) and ABT-263 (99.49% purity) were purchased from Selleck Chem (Houston, TX). A-1210477 was used at a concentration of 5 μM while ABT-263 was used at a concentration of 3.3 μM for all the assays unless otherwise mentioned. Mdivi-1 (98.75% purity) was purchased from MedChem Express (Monmouth Junction, NJ, USA). Drug treatment time was 48 h for all assays except for the Mdivi-1 experiments (Fig. 8b, c). For the triple combination experiments, the treatment time was 24 h for all compounds.

### Cell lines and patient samples

A375, 1205Lu, SK-MEL 28, and HT144 lines each have the *BRAF*^*V600E*^ mutation. WM852c and SK-MEL-2 have the *NRAS*^*Q61R*^ mutation. Hs852T have the *NRAS*^*G12V*^ mutation. PIG1 is an immortalized melanocyte line kindly provided by Dr. Le Poole^33^

Patient samples were derived from melanoma biopsy samples of patients relapsed from various treatments including anti-PDI therapy (Supplementary Table 1). The patient samples either harbored a *BRAF* mutation (MB2195 and MB3429), *BRAF* Fusion (MB1692 and MB1374), *NRAS* mutation (MB3961 and MB3443), or were triple-WT ((MB2046 and MB2141). These melanoma cultures were validated by the University of Colorado skin cancer biorepository with Melanoma Triple Cocktail staining^34^. All patient sample lines were STR profiled and matched >80%.

### ATP viability assay, cell death assay, Annexin V-FITC apoptosis assay, primary and secondary sphere assays

Cell viability was measured and quantified by using the ATP assay (Promega Corp., Madison, WI). Annexin V-FITC Apoptosis Detection Kit (BD Biosciences, San Jose, CA) was used to quantify apoptosis by flow cytometry, according to the manufacturer’s protocol.

All sphere assays were conducted similarly as described in our previous publications^19–21^. A schematic of the experimental layout for the primary and secondary sphere assays is provided in our previous studies^19^. At least three repeats of both the primary and secondary sphere assays were done for each cell line/tumor sample. Drug treatment started on day 5 after seeding for primary sphere assays and 24 h after seeding for monolayer ATP assays.

### Immunoblot

All cells, floating and adherent, were collected and lysed using 2× laemmli buffer (Bio-Rad, Hercules, CA). Samples were used in the standard western blot analysis protocol as described previously^35,36^. The following antibodies were used at suggested dilutions from the manufacturers: PARP, DRP-1, BFL-1, Caspase 3, and α/β TUBULIN were from Cell Signaling Technology (Danvers, MA); NOXA and BIM were from EMD Biosciences, Inc. (San Diego, CA); MCL-1 was from BD Biosciences (San Jose, CA); and HRP-conjugated goat anti-mouse and anti-rabbit antibodies were from Cell Signaling Technology (Danvers, MA).

### Creation of short hairpin RNA transduced cell lines

Stable cell lines were constructed as previously described using shRNA lentiviral particles from Santa Cruz Biotechnology (Santa Cruz, CA) according to the manufacturer’s instructions^35^.

### Creation of CRISPR/Cas9-mediated knockout cell lines

BCL-2 family member BIM was knocked out by CRISPR/Cas9 technology, using a previously published protocol^37^. Briefly, the cells were first subjected to Cas-9 lentiviral transduction and then selected for Blasticydin resistance for 5 days. The Blasticydin-resistance Cas-9 transduced cell lines were then subjected to BIM gRNA lentiviral transduction. The functional Genomics Core at UC Boulder provided CRISPR/Cas9-related vectors, originally provided by Dr. Feng Zhang’s lab (The Broad Institute and the McGovern Institute of Brain Research at the Massachusetts Institute of Technology)^38^. The two different BIM gRNA sequences of the lenti-guide puro-vectors are GCCCAAGAGTTGCGGCGTAT and CAACCACTATCTCAGTGCAA. After transduction, cells were selected for the stable construct with puromycin. The cells were then seeded in 96-well plates at the density of 1 cell/well using the MoFlo XDP100 Cell sorter by the University of Colorado Cancer Center Flow Cytometry Core. The single cells were clonally expanded and screened for the complete knock-out by western blotting of cell lysates.

### Statistical analysis

All the graphs for ATP assay and sphere-forming assays, as well as all statistical analyses, were created using GraphPad Prism 5 software. Error bar indicates ±SEM. One-way analysis of variance (ANOVA) was used to evaluate if there were any statistically significant differences among all the conditions within each experiment. Tukey post-hoc test was then performed to determine which comparison among the conditions was significantly different. The analyses with *p-*value of 0.05 and below were considered significant.

## Results

### A-1210477 in combination with ABT-263 reduced cell viability and killed melanoma cells

**Fig. 1:**
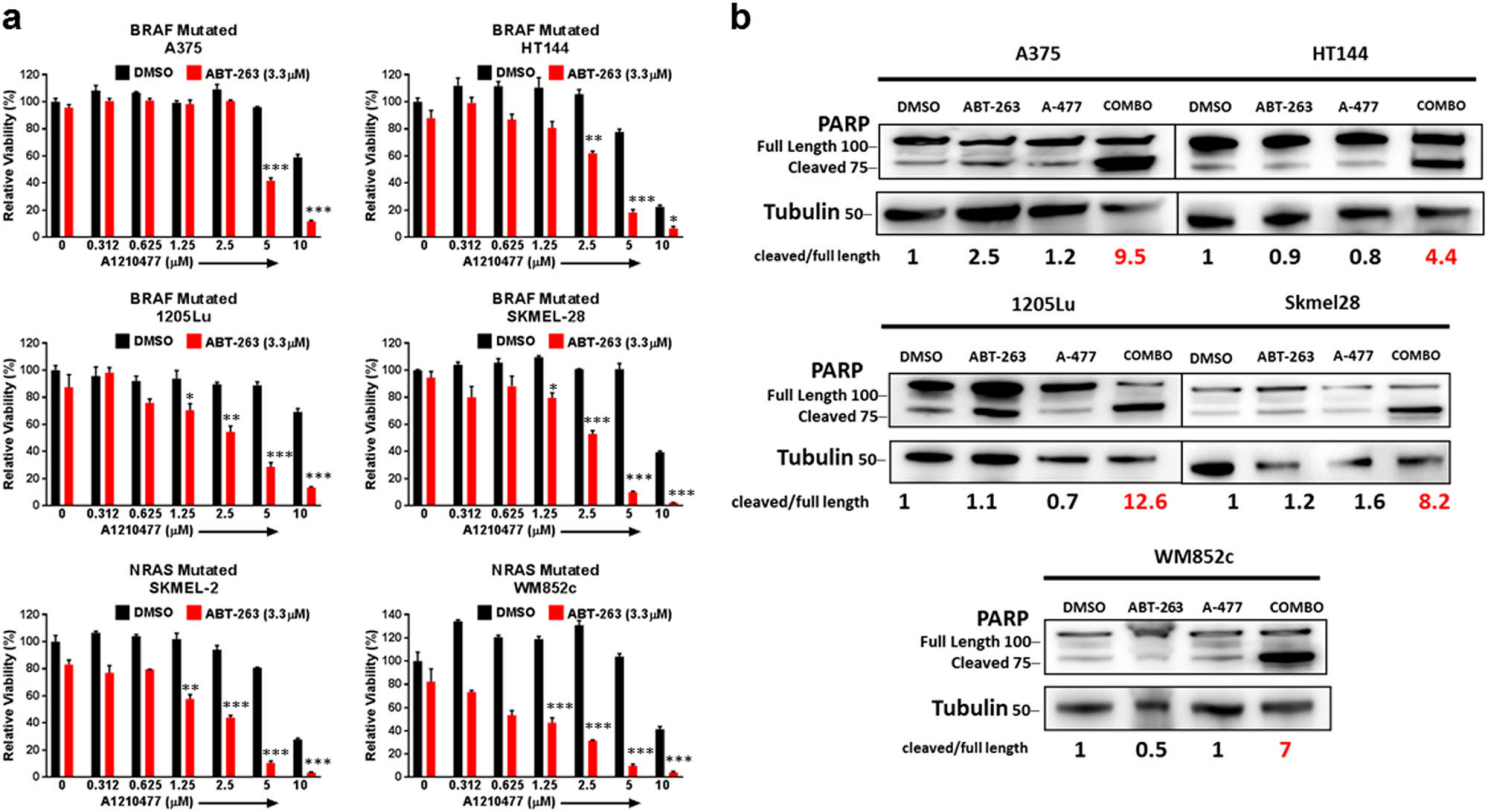
A-1210477 in combination with ABT-263 reduced cell viability and induced apoptosis in melanoma cells, in monolayer conditions. **a** ATP assays of six melanoma cell lines upon indicated treatments for 48 h. The viability of the DMSO control for each cell line was set to 100%. The combination significantly (*p* ≤ 0.01) reduced cell viability compared with DMSO or with single drug treated conditions in all melanoma cell lines. For clarity, we marked only the combinational treatments that were significantly different from comparisons with the DMSO and the single drug treatments. Within each significant combination treatment, we only show the least significant *p*-value of the comparisons. * indicates *p* < 0.05; ** indicates *p* < 0.01; *** indicates *p* < 0.001. **b** Cells were treated for 48 h of control (DMSO), 5 μM A-1210477, 3.3 μM ABT-263 or the combination of 5 μM A-1210477 plus 3.3 μM ABT-263, and then subjected to Immunoblot with an antibody recognizing full length and cleaved PARP. The combination treatment increased the cleaved: full-length PARP ratio in all lines. Molecular weight markers are in kDa

We evaluated the efficacy of combining two BH3 mimetics, A-1210477 plus ABT-263, as an alternative treatment option in melanoma. We chose the maximum dose for each drug that does not have significant effect by itself in multiple melanoma cell lines (data not shown). The combination significantly (*p* ≤ 0.05) reduced cell viability compared to dimethyl sulfoxide (DMSO) or single drug treated conditions in multiple cell lines (Fig. 1a). Visually, the rounded morphology or complete detachment of cells in the combination treated plates, relative to the single drug treatments or control, suggested that the combination induced killing (Supplementary Figure 1). Additionally, we analyzed protein lysates for cleavage of PARP, a well-known marker of cells undergoing apoptosis^39^. The combination treatment induced higher PARP cleavage relative to other treatments. Results were consistent for all the melanoma cell lines tested, irrespective of the mutation status (Fig. 1b). To further determine if killing was at least partially through apoptosis, we utilized Immunoblots for active Caspase 3 (cleaved Caspase 3) and the Annexin V/PI assay (Supplementary Figure 2a, b). Indeed, both assays confirmed that the combination induced apoptosis regardless of the mutation status.

### The combination killed the MICs in multiple melanoma cell lines

**Fig. 2:**
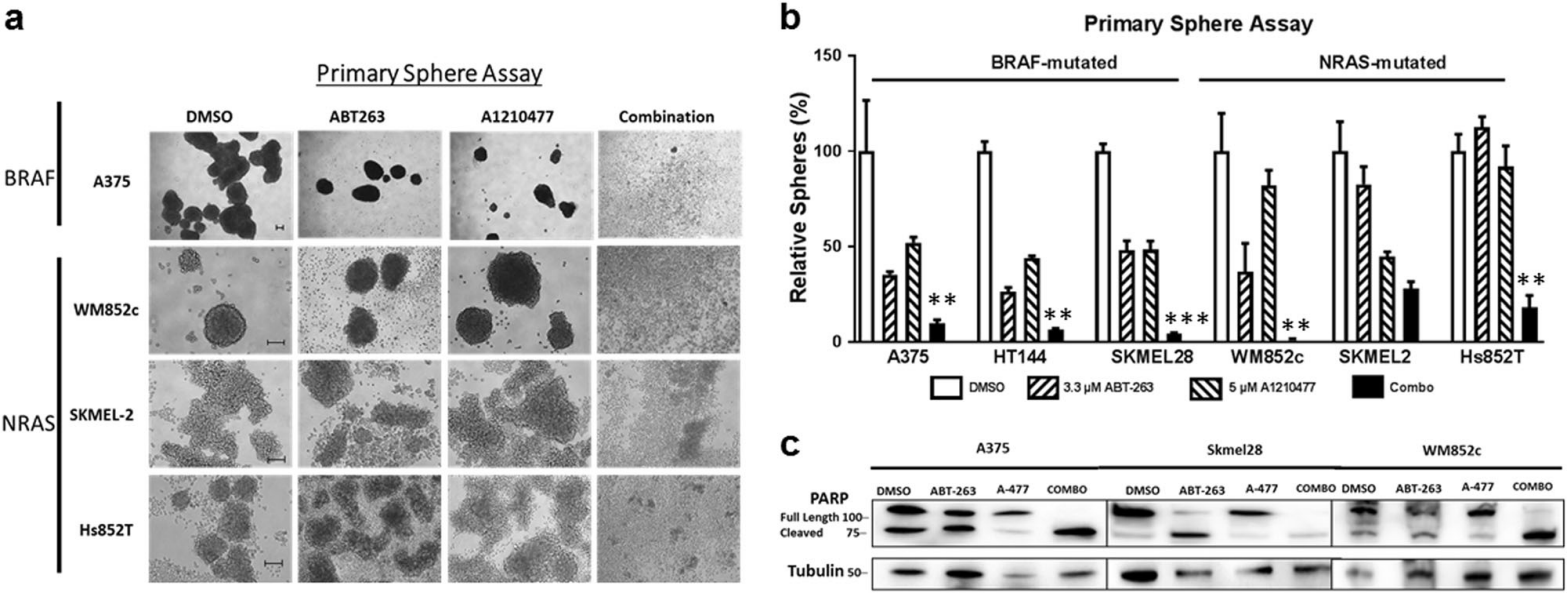
A-1210477 combined with ABT-263 killed the MIC population of melanoma cells regardless of the mutation status. Melanoma cells were subjected to the primary sphere assay. Spheres were treated with indicated compounds either alone, or in combination, of the same concentration as in Fig. 1b for 48 h, and were then subjected to **a** bright field analysis, scale bar = 100 μm and **b** quantification of the number of primary spheres. For clarity, we marked only the combinational treatments that were significantly different from comparisons with the DMSO and the single drug treatments. Within each significant combination treatment, we only show the least significant *p*-value of the comparisons. ** indicates *p* < 0.01; *** indicates *p* < 0.001. **c** Protein lysates were prepared under the same treatment conditions and were probed for PARP. Molecular weight markers are in kDa

Cancer stem cells (CSCs) play a role in cancer relapse, and their elimination is believed to be crucial in cancer treatment^40,41^. The primary sphere assay is one of the best in vitro methods to study CSCs^42^, used to enrich the CSC of melanoma, MICs, and to test drug potency^41–44^. The combination severely disrupted the primary spheres compared to the DMSO or both single drug treatments, in five out of the six melanoma cell lines (Fig. 2a, b). In SK-MEL-2, the effects from the combination was not marked as significant since it was only significantly different from that of the DMSO and ABT-263, but not from A-1210477. Immunoblot showed that the combination treatment increased the PARP cleavage relative to other treatments in the primary sphere lysates (Fig. 2c). Together, these data suggest that the combination treatment is more potent than single drugs in killing the MICs in most of melanoma tested, and the mutation status of *BRAF* or *NRAS* did not influence the effects.

### The combination inhibited the self-renewability of MICs in multiple melanoma cell lines

**Fig. 3:**
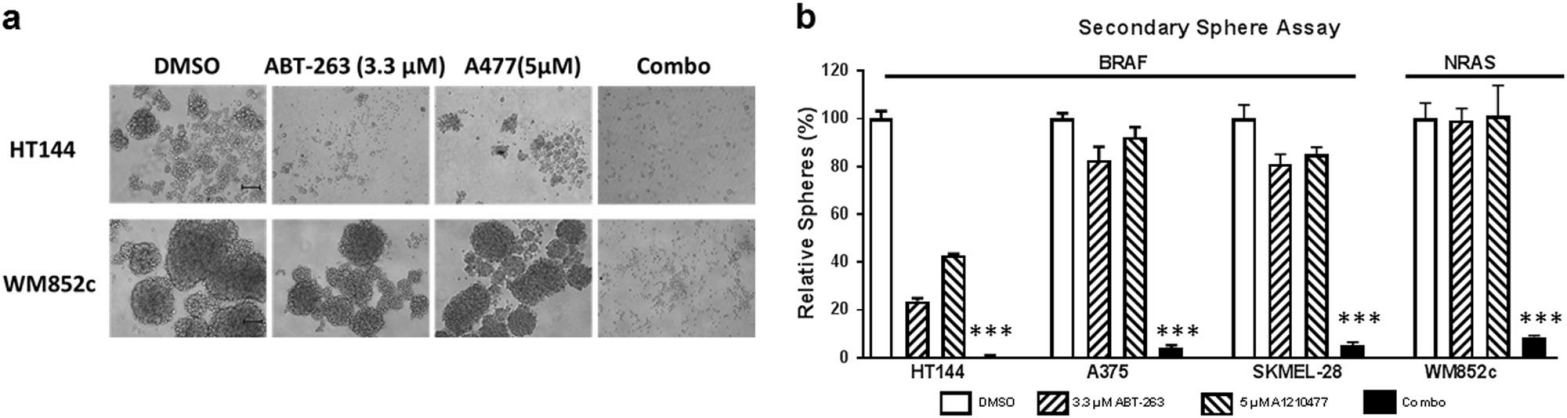
A-1210477 combined with ABT-263 inhibited the self-renewability of MICs. The secondary sphere assay was performed with the indicated melanoma cell lines. **a** Bright field images of secondary spheres. Scale bar = 100 μm. **b** Quantification of the number of secondary spheres. For clarity, we marked only the combinational treatments that were significantly different from comparisons with the DMSO and the single drug treatments. Within each significant combination treatment, we only show the least significant *p*-value of the comparisons. *** indicates *p* < 0.001

The self-renewal capacity of CSCs further contributes to cancer relapse;^45^ preventing self-renewability is necessary to prevent cancer reoccurrence. We utilized the secondary sphere formation assay, a well-known in vitro assay for analyzing self-renewal capacity^43,44^, and tested the treatment effects. The combination treatment eliminated almost all secondary sphere formation (Fig. 3a) compared to the DMSO, ABT-263, or A-1210477 single drug treatments (*p* ≤ 0.001) in all four cell lines tested, irrespective of their mutation status for *BRAF* or *NRAS* (Fig. 3b). Results suggest that the combination decreases MIC’s self-renewal capability.

### The combination was effective in de-bulking and killing MICs of the relapsed patient samples

**Fig. 4:**
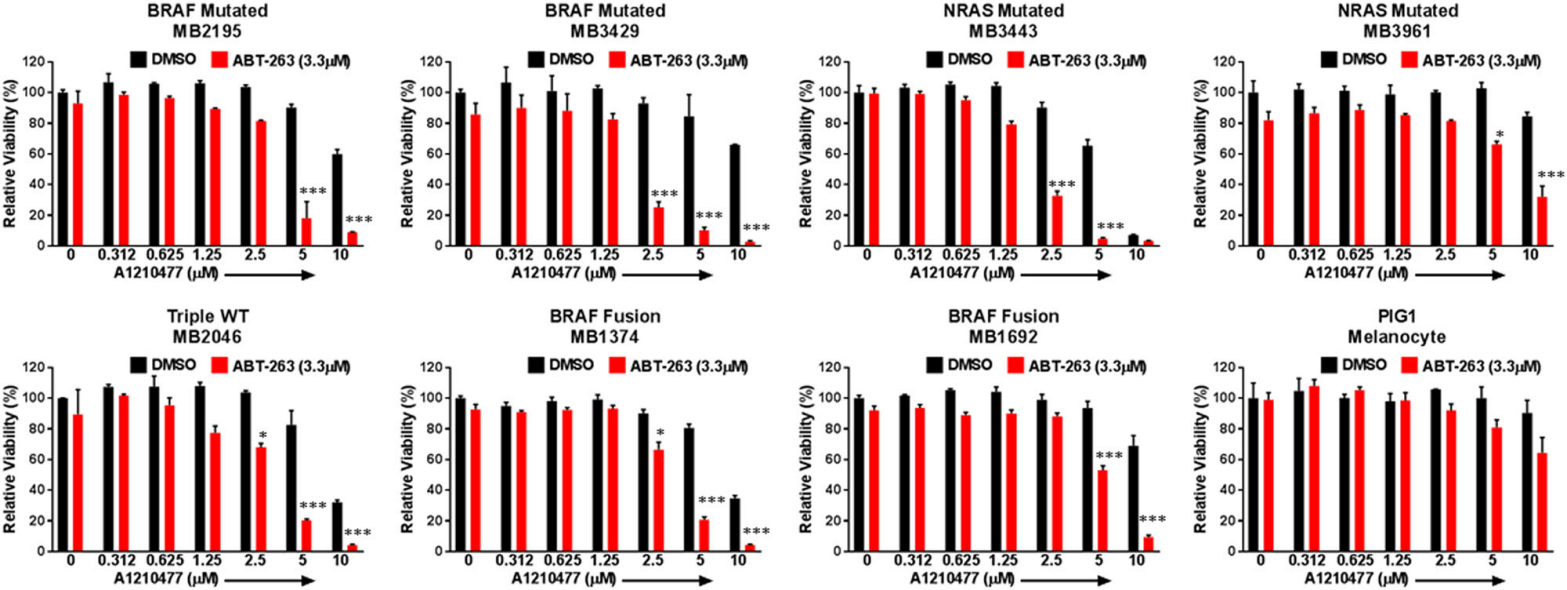
The combination reduces cell viability in multiple relapsed patient samples, but not in normal melanocyte in monolayer culture conditions. ATP assays of melanoma patient samples and melanocytes with indicated treatments. The viability of the DMSO control for each cell line was set to 100%. The combination significantly (*p* ≤ 0.05) reduced cell viability compared with DMSO or with single drug treated conditions in all melanoma cell lines. For clarity, we marked only the combinational treatments that were significantly different from comparisons with the DMSO and the single drug treatments. Within each significant combination treatment, we only show the least significant *p*-value of the comparisons. * indicates *p* < 0.05; *** indicates *p* < 0.001

To assess how effective this combination is in patient backgrounds, we employed multiple short-term monolayer cultures of patient samples. These include samples carrying mutations of *BRAF, NRAS*, or *BRAF*-Fusion, as well as a sample of triple-WT (wild type for *BRAF*, *NRAS,* and *NF-1*). Some of the samples used in the study have relapsed from BRAF/MEK inhibitors or anti-PD-1 immunotherapy treatments (Supplementary Table 1). Importantly, we found that the combination significantly (*p* ≤ 0.05) reduced cell viability compared to DMSO or either single drug treatment, in all the seven melanoma samples tested (Fig. 4). However, the combination treatment only had modest effects in the immortalized human melanocyte line PIG1 (Fig. 4). Immunoblot also showed that the combination treatment increased the cleaved product of PARP relative to other treatments (Supplementary Figure 3). These data suggest that this combination could be clinically relevant with a potent MCL-1 inhibitor, and is a viable treatment approach to further explore for melanoma patients.

**Fig. 5:**
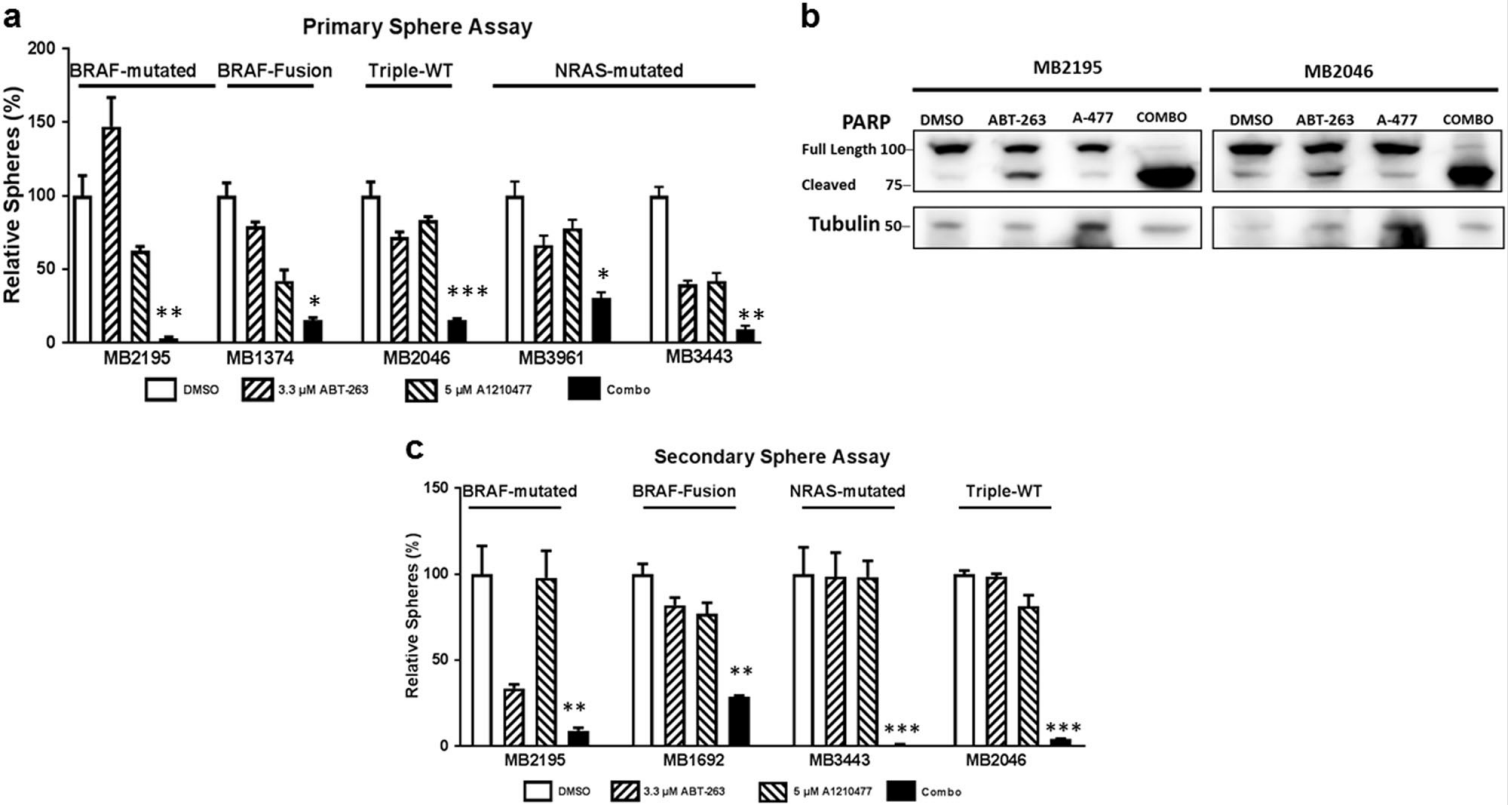
The combination killed the MIC population in multiple relapsed patient samples regardless of the mutation status. Short-term cultures from patient samples were subjected to the primary and secondary sphere assays. Spheres were treated with indicated compounds either alone or in combination, for 48 h. **a** Quantification of the number of primary spheres. **b** Protein lysates were prepared under the same treatment conditions and were probed for PARP. Molecular weight markers are in kDa. **c** Quantification of the number of secondary spheres. For clarity of **a** and **c**, we marked only the combinational treatments that were significantly different from comparisons with the DMSO and the single drug treatments. Within each significant combination treatment, we only show the least significant *p*-value of the comparisons. * indicates *p* < 0.05; ** indicates *p* < 0.01; *** indicates *p* < 0.001

We also examined whether the combination treatment effectively killed the MICs (primary sphere assay) and inhibited the self-renewability of MICs (secondary sphere assay) of relapsed patient samples. The combination treatment severely disrupted the primary sphere formation and significantly reduced the number of primary spheres compared to DMSO and single drug treatment (*p* ≤ 0.05) (Fig. 5a). Immunoblot also showed that the combination treatment increased the cleaved product of PARP relative to other treatments in the sphere lysates (Fig. 5b). In all four samples, the combination treatment also significantly inhibited the formation of secondary spheres compared with DMSO, ABT-263, or A-1210477 treatments alone (*p* ≤ 0.01) (Fig. 5c). Overall, the data suggest that the combination treatment is effective in inhibiting the MICs of relapsed patient samples irrespective of the mutation status (Fig. 5).

### Neither NOXA nor BIM acts as a significant contributor to the combination treatment-induced cell death

**Fig. 6:**
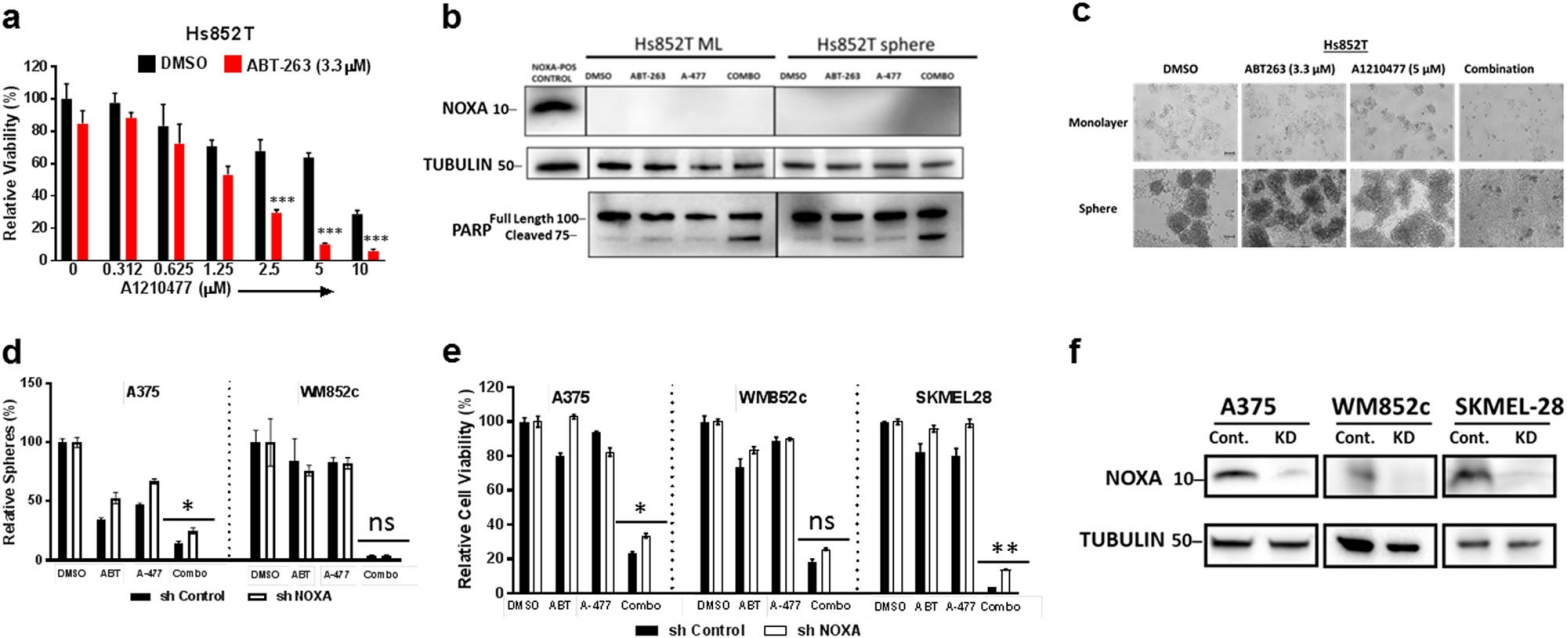
NOXA does not act as a significant contributor to the combination-induced cell death. **a** ATP assay with NOXA null line Hs852T with indicated treatments. **b** Immunoblot with NOXA null line to confirm absence of NOXA. Protein lysates were prepared under the same treatment conditions as in panel **a** before being subjected to western blot and probed for PARP. **c** Monolayer and primary sphere images with the NOXA null line. Primary sphere assay (**d**) or ATP assay (**e**) were performed with the cell lines stably carrying NOXA knockdown (KD) shRNA or control (shControl), upon indicated treatments; For clarity of **a**, we marked only the combinational treatments that were significantly different from comparisons with the DMSO and the single drug treatments. Within each significant combination treatment, we only show the least significant *p*-value of the comparisons. In **d** and **e**, we marked whether NOXA knockdown was significantly different from the shcontrol, upon the combination treatments. * indicates *p* < 0.05; ** indicates *p* < 0.01; *** indicates *p* < 0.001; ns indicates not significant. **f** Western blot confirming the knockdown of NOXA. Molecular weight markers are in kDa

NOXA and BIM play important roles in antagonizing MCL-1 and BCL-2 in melanoma^1,2^, and we have previously found that NOXA and BIM are crucial at inducing cell death when ABT-737 (an earlier version of ABT-263) was used in combination with other agents^2,5,19–21,35,46^. Immunoblots showed that the combination treatment increased the expression of NOXA and BIM in some of the lines (Supplementary Figure 4a and Supplementary Figure 5). However, in a NOXA-null melanoma line Hs852T, the combination treatment was very effective in reducing the cell viability (Fig. 6a, c, upper panel), increasing the cleaved product of PARP (Fig. 6b), and disrupting the primary spheres (Fig. 6c, lower panel). The killing efficacy of the combination was similar to that of wild-type NOXA cell lines tested in Figs. 1 and 2. Thus, NOXA is not necessary for inducing cell death when treated with both A-1210477 and ABT-263.

We also established stable cell lines with NOXA knockdown using an shRNA-mediated approach (Fig. 6f)^35^, and found that knockdown of NOXA only provided a small amount of protection against the combination-induced disruption of primary spheres (Fig. 6d) or reduction in cell viability (Fig. 6e) across melanoma cell lines tested. Visually, we did not see any protection from the combination treatment in monolayer or primary sphere condition in the representative A375 line (Supplementary Figure 4b and c). These results were more consistent with the minor alteration of NOXA:MCL-1 ratio upon the combination treatment (Supplementary Figure 4), a readout sometimes used in cancer drug treatments^47,48^.

**Fig. 7:**
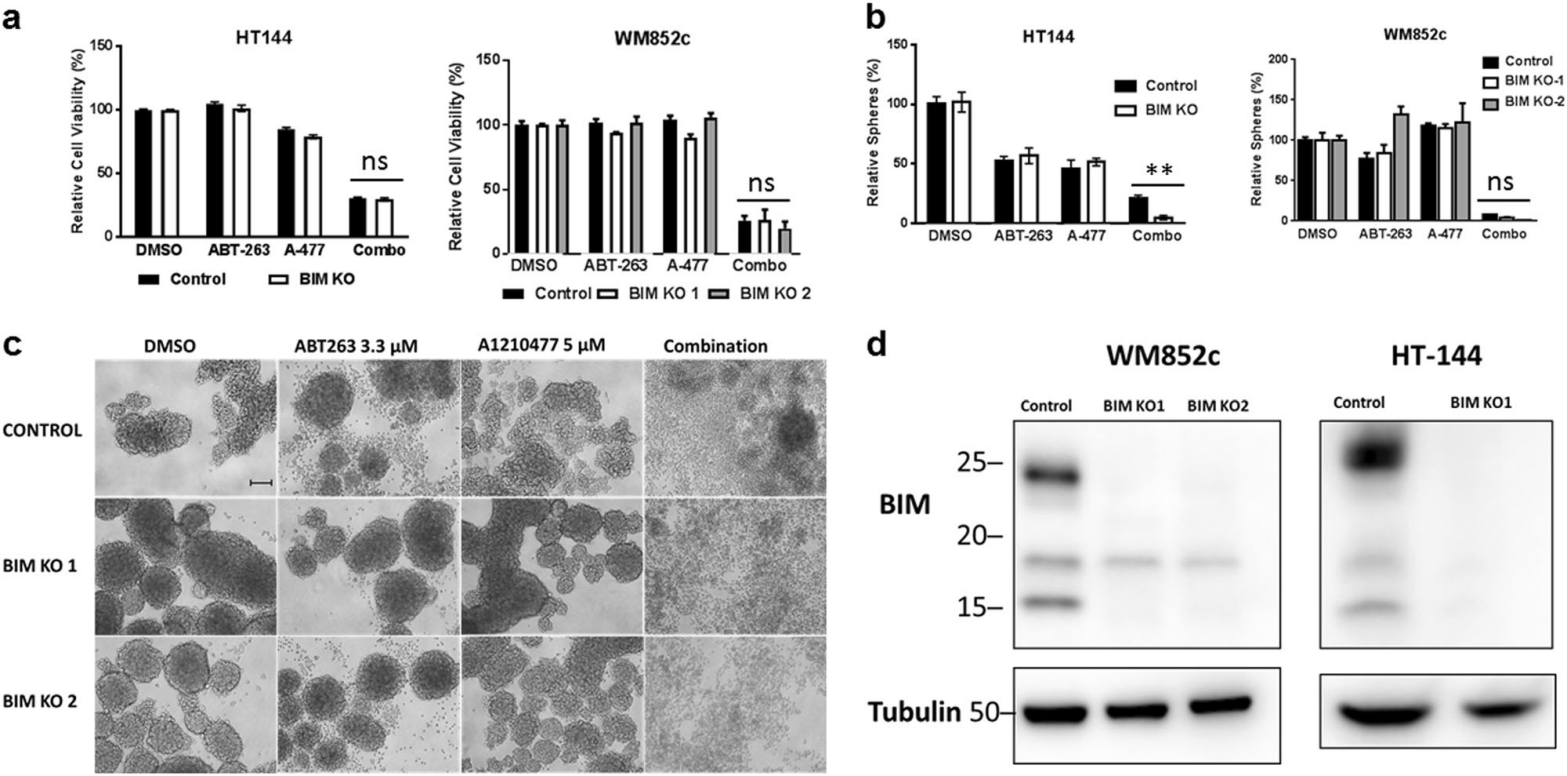
BIM does not act as a significant contributor to the combination-induced cell death. **a** ATP assay and **b** primary sphere assay with BIM KO lines after indicated treatments. For clarity of **a** and **b**, we marked whether BIM KO was significantly different from the control, upon the combinational treatments. Within each significant combination treatment, we only show the least significant *p*-value of the comparisons. ** indicates *p* < 0.01; *ns* not significant. **c** Representative primary sphere images with BIM KO line WM852c after indicated treatment. **d** Western blot confirming the knockout of BIM. Molecular weight markers are in kDa

To dissect the role of BIM, we used the CRISPR/Cas9 technique to successfully knock out BIM (Fig. 7d). Similar to our NOXA results, BIM knockout lines did not consistently protect melanoma cells against the combination-induced reduction in cell viability or primary sphere disruption (Fig. 7a–c). These data suggest that the mechanism driving the combination killing does not require the expression of pro-apoptotic proteins NOXA and BIM.

### The role of BFL-1 in the combination treatment-induced cell death

BFL-1 is an anti-apoptotic BCL-2 family member that is not affected by either A-1210477 or ABT-263. High BFL-1 expression occurs in at least a subset of melanoma and can be an important pro-survival player^49,50^. Therefore, we examined the effects of knocking down BFL-1 on the combination-induced killing, in two cell lines with high endogenous BFL-1 expression (Supplementary Figure 6). Interestingly, in SKMEL-28, knocking down BFL-1 dramatically increased ABT-263-induced cell death, and slightly increased the combination-induced cell death. However, in MB2141, knockdown of BFL-1 seemed to protect cells slightly from treatments of either A-1210477 alone or combined with ABT-263 (Supplementary Figure 6). These results suggest that the effects of BFL-1 are cell-line-dependent, and may not be critical for all the melanoma cells.

### In melanoma, the combination reduces the active form of DRP-1, and DRP-1 inhibition enhances combination-induced cell death

**Fig. 8:**
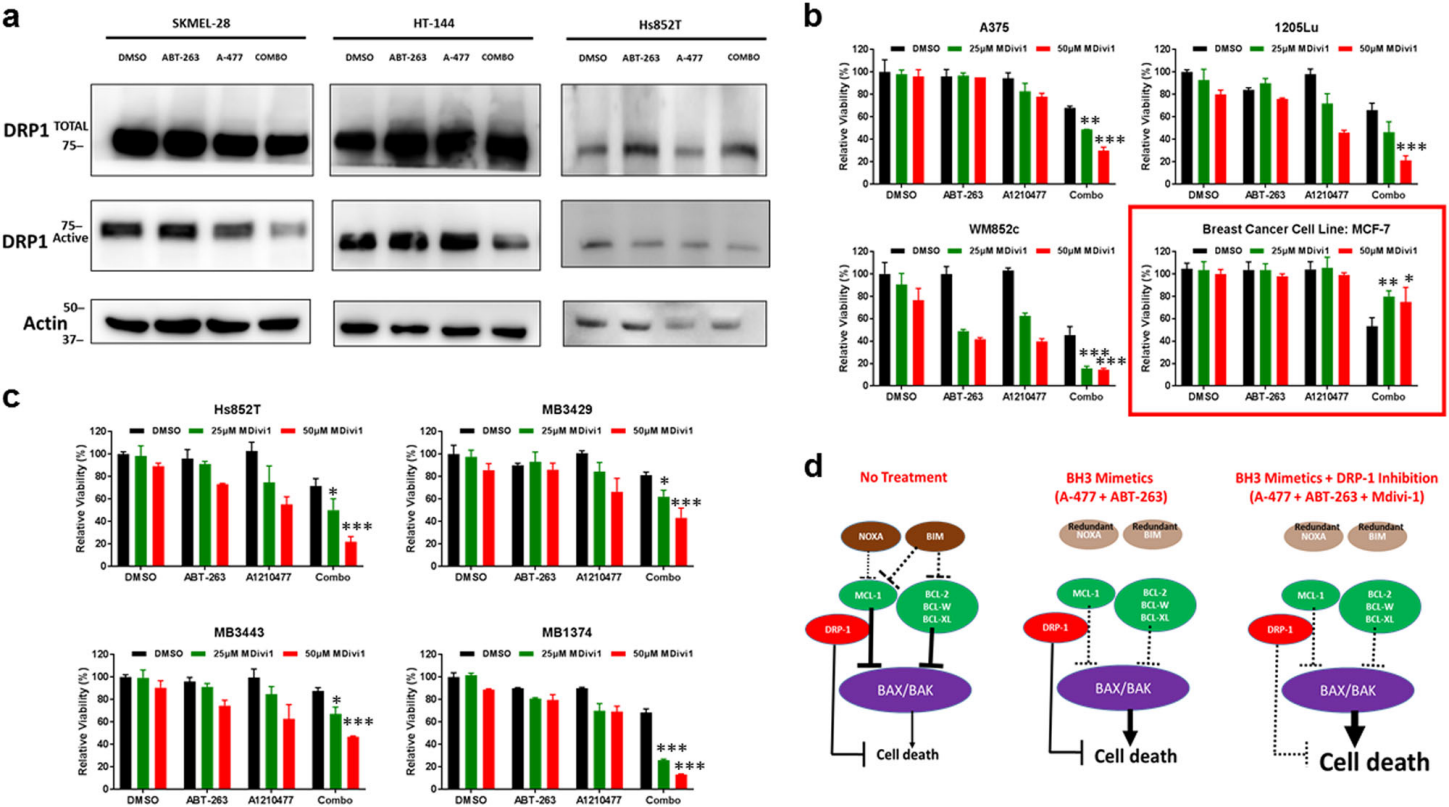
The combination reduces the active form of DRP-1 and induces cell death that is not protected by DRP-1 inhibition in melanoma. **a** Immunoblot showing the active and total DRP-1 in melanoma lines upon indicated treatments. Molecular weight markers are in kDa. **b** ATP assay showing that the pharmacological inhibition of DRP-1 by Mdivi-1 does not protect against cell death in melanoma lines, but protects the breast cancer line MCF-7 (highlighted with red border). **c** ATP assay showing pharmacological inhibition of DRP-1 by Mdivi-1 does not protect against cell death treatment in a NOXA null line or in melanoma patient samples. Treatment time was 24 h for **b** and **c**. For clarity of **b** and **c**, we marked only the triple combinational treatments that were significantly different when compared with the DMSO and the BH3 mimetic combination. Within each significant combination treatment, we only show the least significant *p*-value of the comparisons. * indicates *p* < 0.05; ** indicates *p* < 0.01; *** indicates *p* < 0.001. **d** A simplified melanoma model to illustrate how the treatments induce cell death. Normally (no treatment), melanoma cells have an abundance of anti-apoptotic proteins (green), but not enough pro-apoptotic proteins (brown) to induce death. DRP-1 provides additional protection for cells from cell death. The combinational treatment of BH3 mimetic (A-1210477 + ABT-263) inhibits all the major anti-apoptotic proteins and triggers cell death, without needing pro-apoptotic proteins NOXA or BIM. Addition of DRP-1 inhibitor Mdivi-1 to the BH3 mimetic combination releases all the brakes on apoptosis to induce cell death most effectively

DRP-1 is a large GTPase that can interact with some members of the BCL-2 family^51,52^. In breast cancer cells, it has been shown that A-1210477 induced DRP-1-mediated cell death, and inhibition of DRP-1 protected against BH3 mimetics induced cell death^32^. We examined whether A-1210477 in combination with ABT-263 affects the DRP-1 in melanoma, and found that the combination reduced the active (phosphorylated) form of DRP-1 in multiple melanoma lines (Fig. 8a). We also found that a pharmacological inhibitor of DRP-1, called Mdivi-1^53,54^, enhanced the cytotoxic effect of the A-1210477 plus ABT-263 combination treatment in multiple melanoma cells lines (Fig. 8b). Additionally, Mdivi-1 boosted the cytotoxic effect of the combination in short-term culture of melanoma patient samples with different genetic backgrounds (Fig. 8c). The treatment time was 24 h for the ATP assays. However, as observed in a recent paper^32^, inhibition of DRP-1 by Mdivi-1 protected against the combination-induced cell death in the breast cancer line, MCF-7 (Fig. 8b). Thus, DRP-1 acts opposite in melanoma compared to other cancers, and DRP-1 inhibition induced further cell death in melanoma in response to BH3 mimetics.

To further strengthen our findings, we conducted additional mechanistic studies by knocking down DRP-1 with shRNA lentiviral vectors (Supplementary Figure 7). Consistent with the pharmacological inhibition of DRP-1, knocking down DRP-1 enhanced combination-induced cell death (Supplementary Figure 7). This finding also supports DRP-1 inhibition as an adjuvant to BH3 mimetics treatment in melanoma, but more research is necessary to parse out the relevant mechanism or personalized cell-specific killing.

## Discussion

The treatments for melanoma have significantly advanced recently with the development of molecular targeted therapy and immunotherapy drugs. However, alternative options are still in need, especially for the patients who do not respond or relapse. This study is the first to test the effectiveness of A-1210477 (a potent MCL-1 inhibitor) in combination with ABT-263 (the BCL-2/BCL-W/BCL-XL inhibitor) in melanoma. We have utilized multiple melanoma samples with diverse genetic backgrounds, and on unresponsive or relapsed samples, including to anti-PD1 therapy. Usage of these samples made our study highly relevant to the current clinical setting. Our studies suggest that the concept of targeting multiple BCL-2 family members is worthy of exploring further.

The results here also suggest that targeting multiple BCL-2 family members simultaneously is a promising approach to kill the bulk of melanoma cells, and most importantly, also the MICs. We saw this in cell lines and patient samples suggesting this therapeutic strategy may be clinically applicable. In addition, the mechanistic studies show that direct, potent inhibitors against anti-apoptotic proteins eliminate the need of pro-apoptotic proteins NOXA or BIM to induce cell death in melanoma.

Our study also identified a cell type-dependent role of DRP-1 in BH3-mimetic-induced cell death, with the opposite effects in melanoma compared to breast cancer cells. To our knowledge, this finding has not been reported before. Collectively, our results indicate that both anti-apoptotic BCL-2 family members and DRP-1 contribute to melanoma’s resistance to cell death (Fig. 8d), and BH3 mimetics and DRP-1 inhibitors are promising treatment strategies to be further explored.

Upregulation of anti-apoptotic BCL-2 family members as a resistance mechanism to cell death is one of the hallmarks of cancer. Dysregulation occurs downstream of commonly mutated proteins, including BRAF and NRAS, contributing to the resistance or relapse of targeted therapies. Our experiments with short-term cultures of melanoma patient samples suggest that BH3 mimetics that target multiple anti-apoptotic proteins overcome this resistance. Patient samples with mutations in *BRAF, NRAS*, or *BRAF-*Fusion, as well as triple-WT genetic backgrounds, some who experienced relapse, all showed significant killing effect. Therefore, our results highlight the therapeutic potential of BH3 mimetics for melanoma, worthy of further examination.

Another potential contributor to relapse are CSCs^23,55^. The effectiveness of BH3 mimetics against CSCs is demonstrated in leukemia^56,57^. A higher level of BCL-2 has been reported in breast and colon CSCs^58^, and BCL-XL promotes survival of colon^59^ and lung CSCs^60^. Treatment with ABT-737 killed^60^ or sensitized these cells to chemotherapies^59^. MICs are the subpopulations of melanoma cells with the enhanced ability to initiate, maintain, and resist treatments^17,18,22,43,44,55,61^. Melanoma spheres display stemness, self-renewal capacity, and tumorigenicity^43,44^. In vitro, the primary sphere assay selects for the stem-like population, and the secondary sphere assay measures the self-renewability^19–21,41–44^. Our sphere assay data indicate that inhibition of multiple pro-survival BCL-2 family proteins is also a promising approach to target this resistant population in melanoma.

ABT-263 has been used in multiple preclinical studies^62,63^ as well as in clinical trials^11,12,64^. ABT-263 and its earlier version ABT-737 have demonstrated single-agent efficacy against leukemia and lymphoma, but is ineffective in many solid cancers^65^, including melanoma. Melanomas overexpresses multiple anti-apoptotic BCL-2 family members, and in particular, MCL-1 plays a crucial role in promoting resistance^24–29^. Due to the unavailability of specific and potent MCL-1 inhibitors, researchers have previously partnered ABT-263/ABT-737 with compounds inducing the pro-apoptotic protein NOXA that works by selectively inhibiting MCL-1. In those contexts, others and we have found that combination-induced cell death was always dependent on NOXA, and on BIM in certain instances^19,21,28,62,63^. Here, our result suggest that the combination of ABT-263 with a direct and potent MCL-1 inhibitor is a promising approach for treating melanoma. Future studies are warranted to test the effects of combining ABT-263 with the more potent and clinical-grade MCL-1 inhibitors, such as S63845 (refs. ^66,67^) in melanoma. The recently developed new generation of MCL-1 inhibitors S63845/S64315 is currently in clinical trial for hematological malignancies^68^.

The efficacy of the combination of ABT-263 and A-1210477 has been tested before in non-melanoma cancers^31,69^; however, the mechanism of combination-induced cell death has not been analyzed. We utilized multiple approaches to investigate the contribution of NOXA and BIM, including the CRISPR/Cas9, shRNA techniques, as well as a NOXA-null line. Our results indicated that the direct inhibition of multiple anti-apoptotic proteins eliminates the need of pro-apoptotic proteins of NOXA and BIM. These results are consistent with the Displacement Model of cell death, which states that apoptosis can be triggered without BH3 only activators of apoptosis when the major anti-apoptotic BCL-2 family members are inhibited at once^70,71^. This hypothesis implies that a cell is primed for apoptosis but is held in check by anti-apoptotic proteins; inhibiting anti-apoptotic BCL-2 proteins sends the cellular machinery to its default death pathway. The Displacement Model is seen in several cell types, including cancer cell lines. In colorectal carcinoma cells, BIM, BID, or PUMA are not essential for activating apoptotic pathways, if multiple anti-apoptotic BCL-2 proteins are neutralized^72^. In mouse embryonic fibroblasts, the auto-activation of BAX and BAK can occur through downregulation of BCL-2, BCL-XL, and MCL-1, independent of activation of BH3 only proteins^73^. This suggests that a tumor’s lack of expression of BH3 pro-apoptotic proteins would not prevent them from responding to the combination of ABT-263 and MCL-1 inhibitors. Thus, this combination is promising for hard to treat cancers.

DRP-1 may interact with multiple members of the BCL-2 family, including MCL-1, BAX, or BAK^51,52^; however, the exact relationship is unclear and may be cell type dependent. Milani et al.^32^ found that DRP-1 is required for the effective killing induced by A-1210477 in lung, cervical, and breast cancer cell lines, whereas other studies show DRP-1 contributes to resistance, which has been seen in melanoma^54,74^. Interestingly, higher DRP-1 expression is found in melanoma versus benign nevi, and the DRP-1 inhibitor Mdivi-1 induces dose-dependent cell death in melanomas^54^. In mouse embryonic fibroblasts, DRP-1 activation leads to oncogenic transformation, and genetic or pharmacological inhibition of DRP-1 reverses the effects^74^. In brain tumors, DRP-1 activation contributes to the aggressiveness of tumor-initiating cells, and targeting DRP-1 using RNA interference or Mdivi-1 blocked tumor growth^75^. Our data indicate that BH3 mimetics of A-1210477 plus ABT-263 inhibits the activation of DRP-1, and genetic downregulation or pharmacological inhibition of DRP-1 further enhances the combination-induced cytotoxicity in melanoma (Supplementary Figure 7 and Fig. 8). In the context of melanoma, inhibition of DRP-1, in combination with BH3 mimetics, offers a potential robust treatment plan against resistant or relapsed tumors.

Although the success of immunotherapies in melanoma treatment is remarkable, the side effects highlight the importance of considering how new drugs or drug combinations will affect the immune system. The members of the BCL-2 family are important for functions and survival of various immune cells, and different BCL-2 family members seem to have different roles in specific immune cell types^76^. Therefore, different BH3 mimetics are likely to have various effects on the immune system, depending on their specific targets. For example, ABT-737 (an inhibitor of BCL-2/BCL-XL/BCL-W) reduces the severity of autoimmune diseases in animal models, likely through inducing apoptosis in lymphocytes^76^. This killing effect likely preferentially targets conventional T cells leading to enrichment of the regulatory T cell population^77^, which may decrease anti-tumor immune response. On the other hand, MCL-1 is important for the regulatory T cell population, and GX15-070 (a pan inhibitor of MCL-1/BCL-2/BCL-XL/BCL-W/BFL-1) preferentially induces cell death in the regulatory T cell population and enhanced clearance of lung cancer cells when combined with vaccine^78,79^. Thus, before deciding on the strategies, dosage, and timing of BH3 mimetic treatments, studies are warranted to scrutinize the effects on immune cells.

The remarkable success of BH3 mimetics in chronic lymphocytic leukemia has brought a lot of excitement to their use in treatments of other cancer types. This study strongly supports the use of BH3 mimetics as alternative treatments for melanoma, and we also identify DRP-1 inhibition as a potent adjuvant. These treatments may be effective against melanoma regardless of the mutation status of *BRAF* or *NRAS*, if the tumors are resistant to targeted treatments, or are in relapse from immunotherapy. Combination of clinical-grade, potent BH3 mimetics against multiple anti-apoptotic proteins should be explored as a promising treatment option for melanomas.

## Electronic supplementary material

Supplementary Table 1: Melanoma Patient Sample Information

Supplementary Figure 1

Supplementary Figure 2

Supplementary Figure 3

Supplementary Figure 4

Supplementary Figure 5

Supplementary Figure 6

Supplementary Figure 7
