# Supplementary material for: BH3 mimetics induce apoptosis independent of DRP-1 in melanoma
Source: Cell Death Dis. 2018 Sep 5;9(9):907. doi: 10.1038/s41419-018-0932-z (PMC6125485; doi:10.1038/s41419-018-0932-z)
Supplement: Supplementary file 1 — Supplementary Table 1: Melanoma Patient Sample Information [file 41419_2018_932_MOESM1_ESM.docx]

**Supplementary Table 1: Melanoma Patient Sample Information**

| **Sample** | **Mutation** | **Treatment details of patients** | |
| --- | --- | --- | --- |
|  |  | **Before sample collection** | **After sample collection** |
| MB2195 | BRAF V600E | None | Ipilimumab |
| MB3429 | BRAF G596C | Targeted BRAF/MEK inhibitor; Ipilimumab relapsed; Anti-PD-1 therapy- Partial response | None |
| MB1692 | BRAF-Fusion | surgery | Ipilimumab relapsed ; Anti-PD-1 therapy- Complete Response |
| MB1374 | BRAF-Fusion | No information available | Ipilimumab relapsed ; Anti-PD-1 therapy- Partial response |
| MB3961 | NRAS Q61K | Ipilimumab relapsed; Anti-PD-1 therapy relapsed | None |
| MB3443 | NRAS Q61H | Anti-PD-1 therapy relapsed | None |
| MB2046 | Triple WT* | surgery | No other treatment information available |
| MB2141 | Triple WT*; EML4-ALK Fusion | Ipilimumab relapsed | No other treatment information available |

Note: Triple WT*= Wild type for BRAF, NRAS and NF-1.
