# Supplementary figures and images for: BH3 mimetics induce apoptosis independent of DRP-1 in melanoma
Source: Cell Death Dis. 2018 Sep 5;9(9):907. doi: 10.1038/s41419-018-0932-z (PMC6125485; doi:10.1038/s41419-018-0932-z)

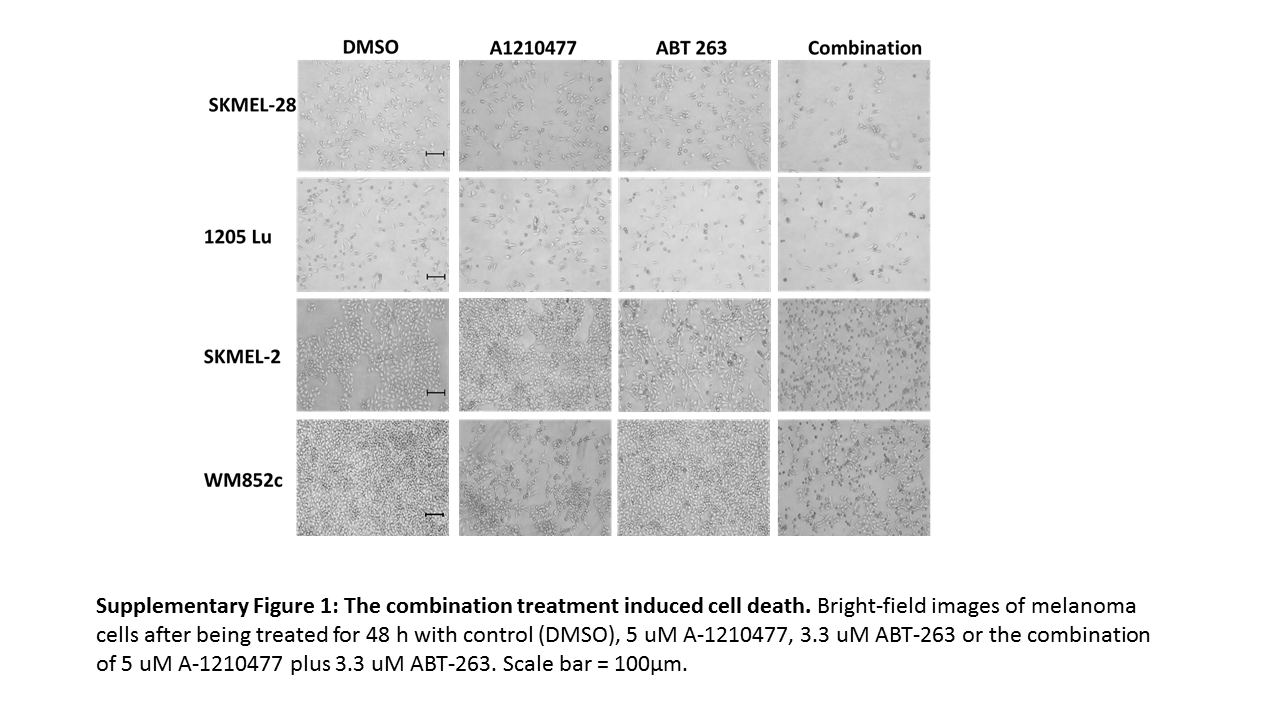

Supplement: Supplementary file 2 — Supplementary Figure 1 [file 41419_2018_932_MOESM2_ESM.tif]

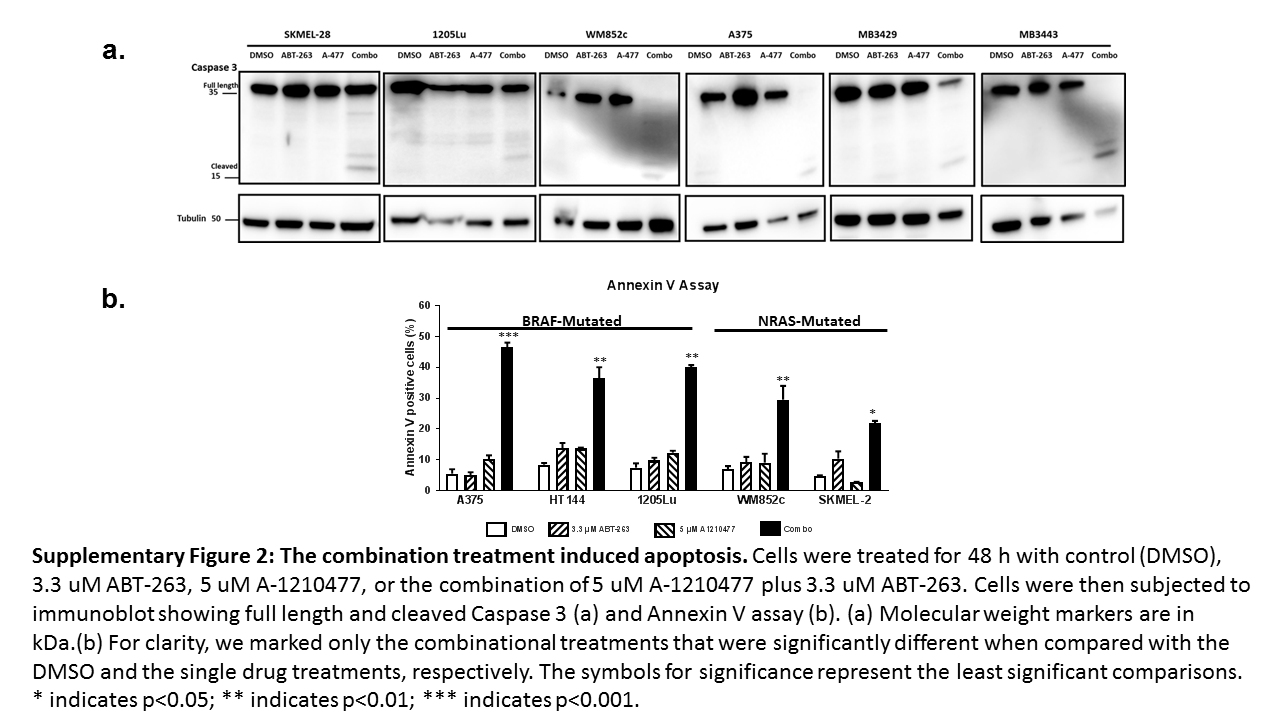

Supplement: Supplementary file 3 — Supplementary Figure 2 [file 41419_2018_932_MOESM3_ESM.tif]

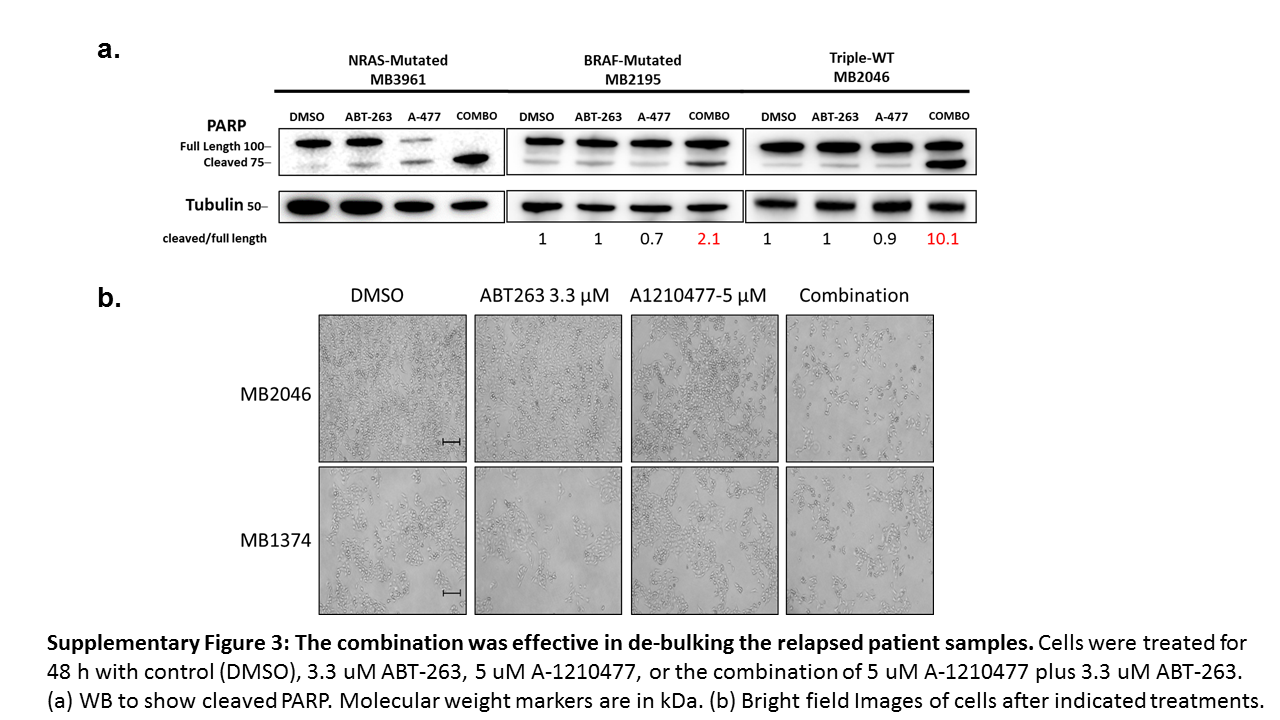

Supplement: Supplementary file 4 — Supplementary Figure 3 [file 41419_2018_932_MOESM4_ESM.tif]

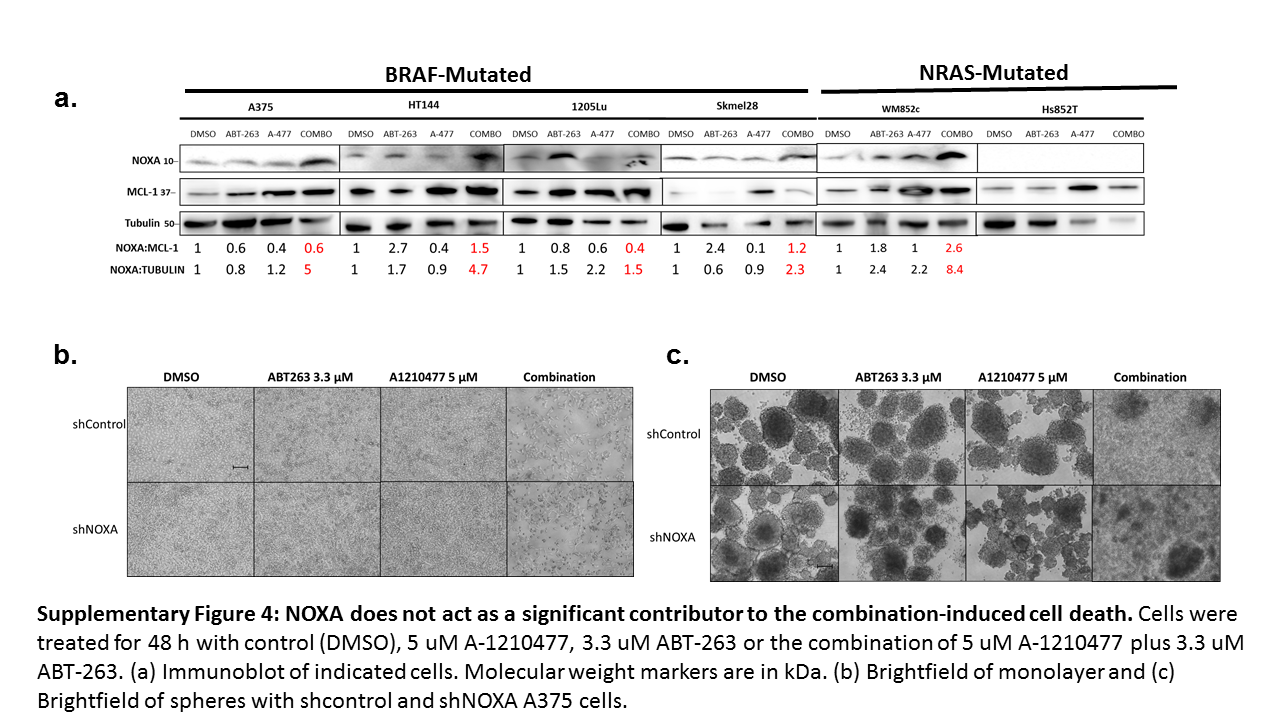

Supplement: Supplementary file 5 — Supplementary Figure 4 [file 41419_2018_932_MOESM5_ESM.tif]

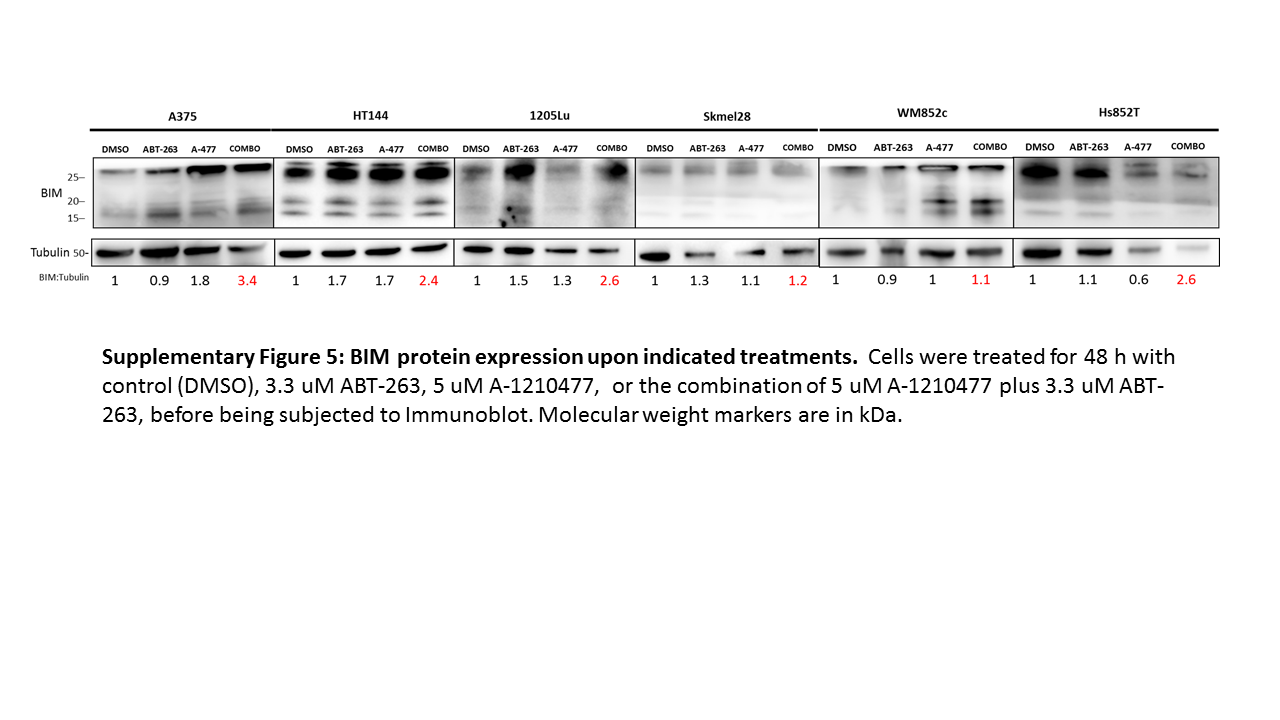

Supplement: Supplementary file 6 — Supplementary Figure 5 [file 41419_2018_932_MOESM6_ESM.tif]

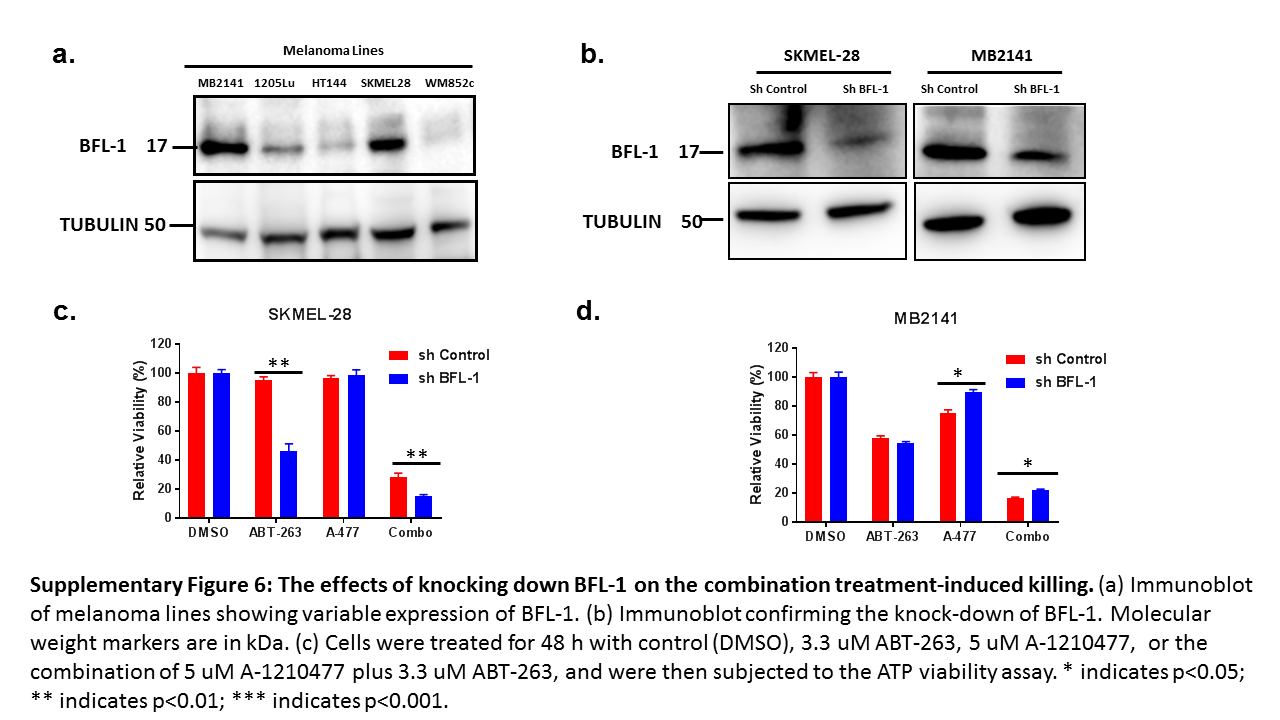

Supplement: Supplementary file 7 — Supplementary Figure 6 [file 41419_2018_932_MOESM7_ESM.tif]

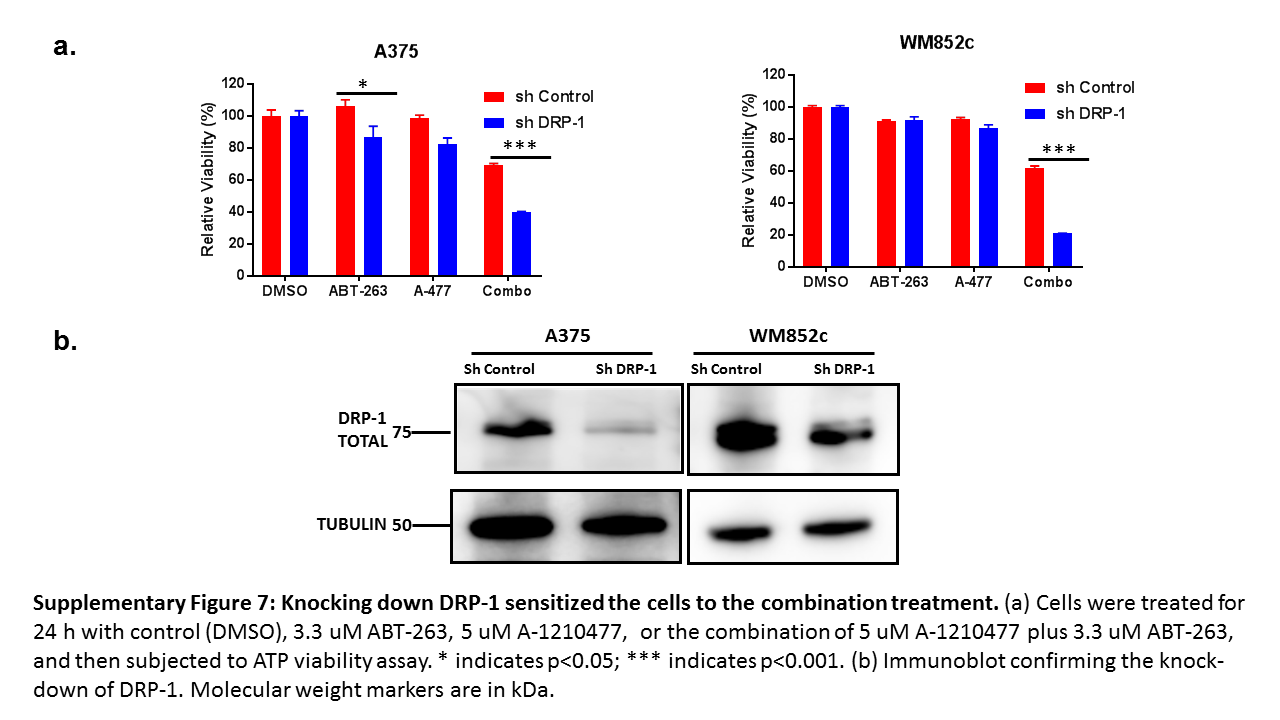

Supplement: Supplementary file 8 — Supplementary Figure 7 [file 41419_2018_932_MOESM8_ESM.tif]
